# Transient Hemi-paresthesia after Eating Puffer Fish (Fugu): A Case Report

**DOI:** 10.7759/cureus.6507

**Published:** 2019-12-30

**Authors:** Hiroki Nagasawa, Ikuto Takeuchi, Kei Jitsuiki, Youichi Yanagawa

**Affiliations:** 1 Acute Critical Care Medicine, Juntendo University Shizuoka Hospital, Izunokuni, JPN

**Keywords:** fugu, intoxication, hemi-paresthesia

## Abstract

The patient was a 64-year-old man who felt numbness of the tongue 30 minutes after eating puffer fish (fugu) prepared by an unqualified person. He then felt hotness on the left side of his face and head, followed by left hemi-paresthesia. The patient had obesity and dyslipidemia. On arrival at our hospital, 150 minutes after eating the fugu, his consciousness was clear, and his only abnormal vital sign was mild hypertension. At approximately four hours after eating the fugu, his hemi-paresthesia spontaneously subsided. He was admitted to our hospital and his post-admission course was uneventful. Brain magnetic resonance image revealed no specific findings. He was discharged on 2^nd^ day of hospitalization without complaint. We presented the 1st case of transient hemi-paresthesia after eating fugu. The mechanism underlying the development of hemi-paresthesia may be pure sensory ischemic attack or fugu intoxication due to an asymmetric distribution of sodium channels.

## Introduction

Pufferfish (fugu in Japanese), a Japanese delicacy, contains tetrodotoxin (TTX), a potent neurotoxin, and fatalities are reported in association with its consumption each year. TTX seems to be produced by endosymbiotic bacteria that often seem to be passed down the food chain. TTX inhibits the sodium channels. In Japan, a regulatory limit of 2 mg eq TTX/kg was established [1]. Symptoms of TTX intoxication typically begin within 30 minutes of ingestion and range from mild paresthesia of the tongue and/or extremities, nausea and pain to full paralysis, hypotension, and death from respiratory failure [2]. Recently, the overall mortality rate was reported to be 7%, and epidemiological evidence suggests that those who survive for 24 hours will make a full recovery [1,2]. In Japan, the restaurant preparation of fugu is strictly controlled by law and only qualified chefs are allowed to prepare the fish. We herein report a case of transient hemi-paresthesia after eating puffer fish.

## Case presentation

**Figure 1:**
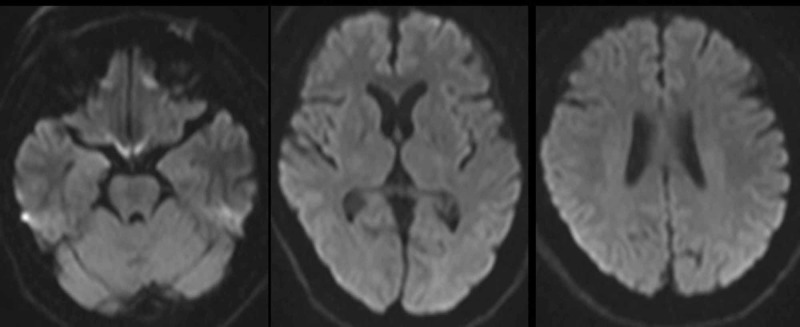
Brain magnetic resonance image (MRI) on the 2nd day of hospitalization (diffusion weighted image). The MRI reveals no specific findings.

The patient was a 64-year-old man who felt numbness of the tongue 30 minutes after eating fugu prepared by an unqualified person. He then felt hotness of the left side of his face and head, followed by left hemi-paresthesia of the left upper and lower extremities. He visited a local medical clinic and was transferred to our hospital with suspected fugu intoxication. He had obesity and dyslipidemia. On arrival, 150 minutes after eating the fugu, his consciousness was clear, his blood pressure was 146/89 mmHg, his heart rate was 65 beats per minute and his percutaneous oxygen saturation was 97% under room air. He complained of left hemi-paresthesia but there were no physical findings. Both electrocardiography and chest roentgenography revealed no specific findings. A venous blood gas analysis revealed the following: pH, 7.40; PCO_2_, 41.8 mmHg; HCO_3_^-^, 26.7 mmol/L; base excess, 1.0 mmol/L; and lactate, 0.9 mmol/L under room air. The main result of a blood analysis was total cholesterol, 261 mg/dL. We did not measure serum TTX level because it could not be measured in our hospital or at any available commercial based biochemical laboratory company. Initially, he refused to undergo a cerebral radiological study, including magnetic resonance imaging (MRI). He underwent gastric irrigation with charcoal and laxative administration. At approximately four hours after eating fugu, his hemi-paresthesia spontaneously subsided and he felt an abnormal sensation on the left side of his head. He was admitted to our hospital for observation. The post-admission course was uneventful. He agreed to undergo brain MRI and MR angiography, which revealed no specific findings (Figure 1). He was discharged on the second day of hospitalization without complaint.

## Discussion

This was the first case of transient hemi-paresthesia after eating fugu. There are two hypothesized mechanisms of the hemi-paresthesia in the present case. One is that it was a symptom of pure sensory stroke, because he had risk factors for arteriolosclerosis, including advanced age, obesity and dyslipidemia. His symptoms may have been due to a transient ischemic attack at the right thalamus [3]. However, brain MRI did not show any evidence of cerebral ischemia in the present case. The second possibility is that he experienced unilateral symptoms due to fugu intoxication because his symptoms were typical of mild fugu intoxication. However, the symptoms of fugu intoxication are generally bilateral. His intoxication was thought to be mild [4]. Some organs or receptor channels might have been responsible for the asymmetric distribution [5, 6]. Accordingly, the incidental asymmetric distribution sodium channels, and mild fugu intoxication might have explained the hemi-paresthesia of the present case. We could not determine which hypothesis was dominant.

Yong et al. also reported a case of asymmetrical symptoms after eating fugu [7]. According to their report, a 35-year-old woman presented with giddiness and weakness of the left upper limb and both lower limbs in addition to numbness and a tingling sensation around her mouth area. These symptoms occurred approximately one hour after consuming fugu at a local Japanese restaurant in Singapore so that they concluded that her symptoms were likely fugu intoxication based only on the medical history without measuring TTX. Her symptoms resolved the following day. Based on their theory, our case might therefore also be fugu intoxication.

## Conclusions

We presented a case of transient hemi-paresthesia that occurred after eating fugu. The mechanism underlying the development of hemi-paresthesia might have been a pure sensory ischemic attack or fugu intoxication due to asymmetric distribution of sodium channels. We could not determine which hypothesis was dominant.
